# Cross-species behavior analysis with attention-based domain-adversarial deep neural networks

**DOI:** 10.1038/s41467-021-25636-x

**Published:** 2021-09-17

**Authors:** Takuya Maekawa, Daiki Higashide, Takahiro Hara, Kentarou Matsumura, Kaoru Ide, Takahisa Miyatake, Koutarou D. Kimura, Susumu Takahashi

**Affiliations:** 1 Graduate School of Information Science and Technology, Osaka University, Osaka, Japan; 2 Graduate School of Agriculture, Kagawa University, Kagawa, Japan; 3 Graduate School of Brain Science, Doshisha University, Kyoto, Japan; 4 Graduate School of Environmental and Life Science, Okayama University, Okayama, Japan; 5 Graduate School of Science, Nagoya City University, Aichi, Japan

**Keywords:** Data mining, Machine learning

## Abstract

Since the variables inherent to various diseases cannot be controlled directly in humans, behavioral dysfunctions have been examined in model organisms, leading to better understanding their underlying mechanisms. However, because the spatial and temporal scales of animal locomotion vary widely among species, conventional statistical analyses cannot be used to discover knowledge from the locomotion data. We propose a procedure to automatically discover locomotion features shared among animal species by means of domain-adversarial deep neural networks. Our neural network is equipped with a function which explains the meaning of segments of locomotion where the cross-species features are hidden by incorporating an attention mechanism into the neural network, regarded as a black box. It enables us to formulate a human-interpretable rule about the cross-species locomotion feature and validate it using statistical tests. We demonstrate the versatility of this procedure by identifying locomotion features shared across different species with dopamine deficiency, namely humans, mice, and worms, despite their evolutionary differences.

## Abstract (web summary)

Comparing changes in behaviour across various species is not always trivial, especial across significantly divergent species. Here, the authors develop a deep learning framework that allows them to map changes in locomotion demonstrated on dopamine-deficient humans, mice and worms.

## Introduction

**Fig. 1:**
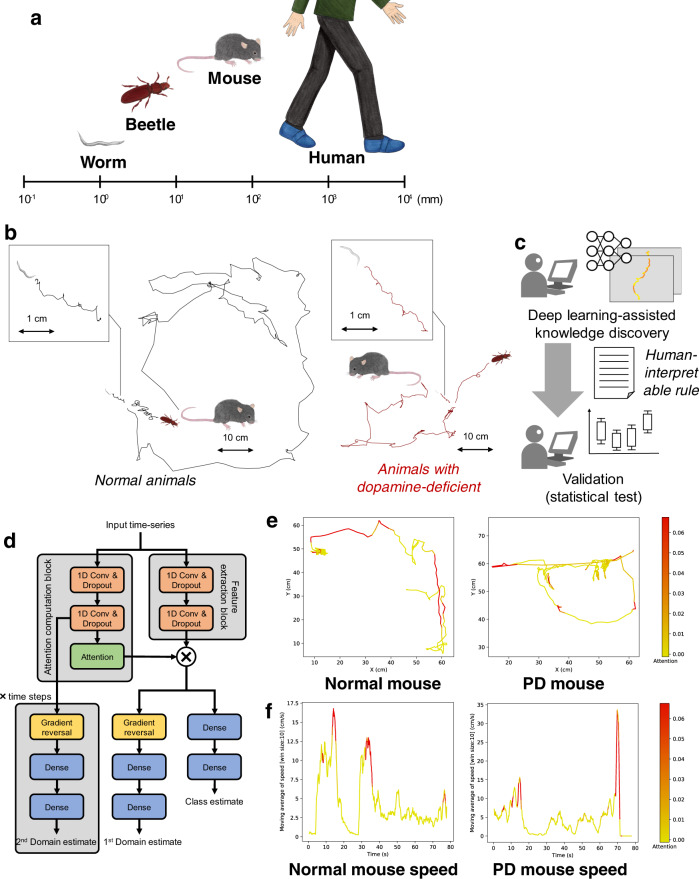
Cross-species behavior analysis using a domain-adversarial neural network with attention mechanism. **a** Differences in body scales among different species. **b** Locomotion trajectories of different animals (worm, beetle, and mouse) differ at the spatial scale, but show similar patterns, left, black lines depict locomotion trajectories of normal animals, right, red lines show trajectories of dopamine-deficient individuals, inset, expanded trajectories of worms. **c** Our proposed procedure automatically finds a locomotion feature shared by different animals using deep learning, and exhibits the learned locomotion features, enabling the human operator to extract a hypothesis and validate the statistical significance. **d** Proposed network architecture: the feature extraction block learns feature representation that maximizes class prediction accuracy but minimizes domain prediction accuracy by using a gradient reversal layer; the learned feature is assumed to be domain-independent because the feature is incapable of distinguishing between the domains, while the attention computation block computes an attention value for each time slice in a domain-independent manner by using a gradient reversal layer. **e** Highlighted trajectories of normal and Parkinson’s disease (PD) mice by attention values of the neural network that is trained to extract cross-species locomotion features of worms and mice. **f** Example time-series of the speed of normal/PD mice highlighted by our network, where the attention level is color-coded according to the scale bar shown on the right.

Neurodegenerative diseases including Parkinson’s disease (PD), Alzheimer’s disease, and schizophrenia are disorders characterized by motor dysfunctions. Since the variables inherent to such diseases cannot be controlled directly in humans, behavioral dysfunctions and their neural underpinnings have been examined in model organisms^1,2^. Assuming that fundamental aspects of the behavior of humans are evolutionarily conserved among other animal species, studies in model organisms gained insights into understanding the underlying mechanisms of those diseases^3–6^. In contrast to cognitive abnormalities, motor dysfunctions can be externally assessed by comparative behavioral analyses. A central concept of comparative behavioral analysis is to identify human-like behavioral repertoires, called behavioral phenotype, in animals, as animal models of PD have gained insight into understanding the behavioral and neural underpinning of symptoms underlying a specific disease^7^ and can potentially provide clues for the development of therapeutics. However, behavioral analysis across animal species could not be realized using conventional statistical analyses because the body scale and locomotion methods vary among species (Fig. 1a), resulting in wide variations in the spatial and temporal scales of locomotion among species as shown in Fig. 1b in which worms, beetles, and mice show similar locomotion trajectory, but differ in the velocity and spatial area occupied.

Deep learning is a novel technique of automated feature extraction, reducing the cost of manual feature design. We employ it for automatically discovering scale-invariant locomotion features shared among animal species. In the procedure (see Fig. 1c), a human operator first feeds locomotion data from animals of different species with different properties into a neural network designed to extract cross-species locomotion features using domain-adversarial training^8^. This is trained to extract features that classify a trajectory into an appropriate class (e.g., healthy or PD) but cannot label the trajectory into an appropriate domain (i.e., species), using a gradient reversal layer as shown in Fig. 1d. Because these features are incapable of distinguishing between domains, we can regard them as species-independent. In contrast, because we can distinguish between trajectories belonging to different classes using the extracted features, these can be regarded as cross-species hallmarks of the diseases. By designing a deep neural network so that it extracts features based on the above idea, we can obtain locomotion features shared across different species independent of their body scales and locomotion methods.

Despite the human-level outstanding performances of the neural network, the human operator cannot understand the meaning of the extracted cross-species locomotion feature by the deep neural network containing a huge amount of hidden parameters. To address this issue, we design an explainable architecture by incorporating an attention mechanism^9,10^ into the domain-adversarial neural network, which identifies segments in the trajectories where the cross-species features are hidden and provides visualized trajectories and time-series of basic locomotion features (e.g., speed) by highlighting the identified segments (Fig. 1e, f). From these highlighted graphs, the operator can understand cross-species locomotion features extracted by the neural network. For instance, short-duration peaks in speed are characteristic to PD mice (Fig. 1f). To explain why the neural network pays attention to a certain segment and how it labels an input time-series using attended segments within the time-series, we employ decision trees. They help formulate a human-interpretable rule about the cross-species locomotion feature. Then, the operator proposes a hypothesis related to the locomotion features and performs a statistical test to validate it (see Fig. 1c).

To demonstrate the performance of our proposed cross-species behavioral analysis, we identified locomotion features shared across different species with dopamine deficiency, namely humans, mice, and worms.

## Results

### Attention-based domain-adversarial neural network

This study assumes that locomotion data from two different species that belong to two different classes, for example, PD individuals and healthy individuals of humans and mice are given. The locomotion data are used to train the neural network (see Fig. 1d). The model inputs are time-series of primitive locomotion features such as speed. The model has two types of outputs: estimated domain and class of an input time-series.

The convolutional layers in the feature extraction block are used to extract features, which are used to output the two estimates. We introduce the gradient reversal layer^8^ before the 1st domain predictor. When we train the network using the backpropagation algorithm^11^, the gradient reversal layer multiplies the gradient with a negative constant value, making the convolutional layers in the feature extraction block incapable of estimating domains but classes.

In addition, we introduce an attention mechanism^9^ into the model. The attention of a data point at each time slice is regarded as the importance of the data point when the input time-series is classified, indicating that attended data points are characteristic to a class to which the input time-series belongs. The convolutional layers in the attention computation block are used to compute attention for each time slice. The attention is multiplied by the extracted features to contrast the data points to which the network pays attention. Furthermore, to make the way of attention computation domain-independent, we introduce the gradient reversal layer after the 2nd convolutional layer in the attention computation block. The 2nd domain predictor outputs domain estimate for each time slice using the output of the 2nd convolutional layer in the attention computation block and we make the network incapable of classifying the output of the 2nd convolutional layer into domains using the gradient reversal layer.

Here we briefly explain the difference between the network and that proposed in DeepHL (DeepHL-Net)^12^, which is our prior work. Because DeepHL-Net is trained on trajectories from a single species, DeepHL-Net outputs only a class estimate, for instance, healthy or PD class. Therefore, DeepHL-Net relies mainly on attention mechanisms that detect segments characteristic of a class. In contrast, in this study, the network is trained on trajectories from two species, and outputs domain and class estimates. To render the network incapable of distinguishing between the two domains, we introduce domain-adversarial training (gradient reversal). More specifically, gradient reversal is introduced to render the ways of feature extraction and attention computation domain-independent.

The network is trained to minimize the error of the class estimates as well as maximize the errors of the two types of domain estimates. However, achieving these conflicting goals at the same time makes it difficult for the neural network to converge. Therefore, we iterate the following procedures to train the network.

1. We first train the network to minimize the error of the class estimates as well as maximize the error of the domain estimates by the 2nd domain predictor, which employs outputs of the attention computation block to predict the domain. Thus we extract important segments in the input time-series for class estimation in a domain-independent manner.
2. Then we update the network to maximize the error of domain estimates by the 1st domain predictor to accelerate training in the next procedure.
3. Finally, we update the network to minimize the error of class estimates as well as maximize the error of domain estimates by the 1st domain predictor. With this procedure, we can train the network to predict class labels correctly but domain labels incorrectly.

See Methods for more details about the procedures.

In addition, to help understand the meaning of attention, we employ machine learning tools to explain it. We construct a decision tree that is trained to detect the attended segments by the neural network using exhaustively handcrafted features listed in Supplementary Table 1.

Furthermore, to interpret how the neural network predicts classes using attended segments, we build a decision tree that is trained to classify an input time-series using handcrafted features extracted only from attended segments within the time-series. See Methods for more details.

As above, an interpretable rule is extracted from our neural network with the explainable architecture and/or by our methods based on decision trees. After that, the interpretable rule is investigated via a statistical test. To validate our procedure, we prepared locomotion data from four different dopamine-deficient species (humans, mice, beetles, and worms). We train our neural network on data from two species to obtain an interpretable rule and then validate this using data from all the species. We only use data from two species because doing otherwise would increase the complexity of the classification task, making it difficult for the network to converge.

We have made the codes for our deep learning method and decision tree construction available as Supplementary Software 1.

### Network training on worm and mouse data

**Fig. 2:**
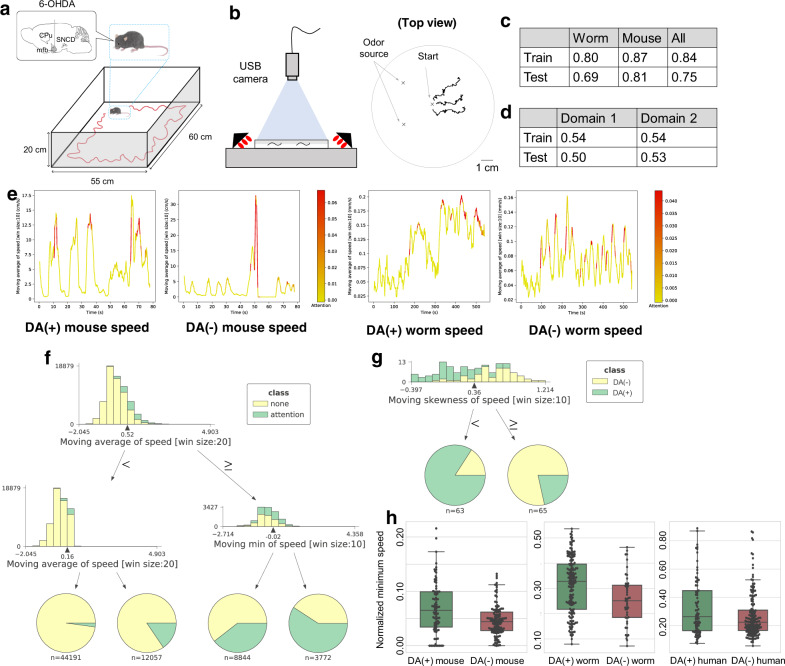
Analysis of DA(+)/DA(−) mice and worms (DA: dopamine). **a** A Parkinson’s disease (PD) mouse walks in the experimental setup, where dopaminergic neurons in the substantia nigra pars compacta were unilaterally lesioned with 6-hydroxydopamine. **b** The experimental setup (left) for monitoring the worm’s trajectory (right). **c** Classification accuracies of our network for DA(+)/DA(−) classes. **d** Classification accuracies of our network for the 1st and 2nd domain estimates, where the random guess ratio is 0.5 (1/2); see Supplementary Information for the detailed results of the domain estimates. **e** Example time-series of speed of DA(+)/DA(−) mice and worms highlighted by our network. **f** A decision tree for explaining the meaning of attention by the neural network, i.e., classifying attended (`attention') and non-attended (`none') segments, where features were normalized for each trajectory (min–max normalization); we show only the 1st and 2nd layers and each histogram shows a tree node of the tree, and a feature used in the node is indicated at the bottom of the histogram; a histogram of each node is constructed from training instances' values of a feature used in the node, instances having feature values smaller than a threshold go to the left child, the threshold value is shown at the bottom of each histogram associated with a black arrow and pie charts at the bottom of the tree (leaf nodes) show distributions of training instances classified by the tree. **g** A decision tree for explaining classification, where we show only the 1st layer. **h** Distributions of averaged minimum speed within a time window when the speed is high for mice/worms; to compute the averaged minimum speed, we compute the rolling minimum of the normalized speed within segments with high speed (top 20% average speed) for each trajectory (see Supplementary Information for details) and the box plot shows the 25–75% quartile, with embedded bar representing the median and the lower/upper whiskers show Q1-1.5*IQR and Q3+1.5*IQR, respectively, where IQR is the interquartile range, Q1 is 25% quartile, and Q3 is 75% quartile; significant differences between DA(+) and DA(−) for both the mice and worms are observed (worm by the Brunner–Munzel test: *p* = 7.61 × 10^−4^: *w* = 3.48; df = 90.41; effectsize = 0.65; *n* = 162 from DA(+) worms; *n* = 47 from DA(−) worms; mouse by the Welch’s *t*-test: *p* = 2.53 × 10^−3^; *t* = 3.07; df = 131.45; effectsize(r) = 0.45; *n* = 88 10 min trajectories from DA(+) mice; *n* = 113 10 min trajectories from DA(−) mice); a significant difference between DA(+) and DA(−) for the humans is also observed by the Welch’s *t*-test (*p* = 0.03; *t* = 2.25; df = 123.83; effectsize(r) = − 0.32; *n* = 78 from DA(+) humans; *n* = 163 from DA(−) humans); the *p* value is two sided; see Supplementary Information for the normality tests of the distributions, which were used to select methods of statistical test for comparing two groups.

We use locomotion data from worms with/without DOP-3, one of the D2 dopamine receptors^13^, and from healthy and PD mice. A PD mouse is a unilateral 6-hydroxydopamine (OHDA) lesioned model of PD whose dopaminergic neurons in the compact part of substantia nigra of a left or right hemisphere are lesioned with a neurotoxin 6-OHDA. Fig. 2a, b show the data collection protocols. Prior studies discovered relevant locomotion features of PD mice^1,2^ as well as of worms during odor avoidance behavior^14,15^. However, locomotion features across these species have not been investigated because the scales of their movements are significantly different. We convert 2D locomotion data from worms and mice into time-series of locomotion speed and then standardize each time-series to normalize the range of the variables. Note that because the lengths of the time-series of the speed of worms differ from those of mice, we have undersampled the time-series from the mice so that the lengths of the time-series are identical to those from the worms. More details about the data are available in Methods and Supplementary Table 2.

We randomly split the speed time-series data into training and test sets, which are fed into the neural network. We deal with two classes, DA(+) class (healthy mice and worms) vs. DA(−) class (PD mice and worms lacking D2 dopamine receptors), and two domains (mouse and worm). The classification accuracy is shown in Fig. 2c, d, indicating that our network cannot distinguish the domains but the classes. These results indicate that cross-species features of DA(−) among mice and worms exist. Fig. 2e shows an example of time-series of speed for worms and mice highlighted by the trained model. From these highlighted time-series, we can speculate that the neural network focuses on segments corresponding to high speed for the DA(+) worms and mice.

Figure 2f shows a decision tree trained to detect the attended segments. The root node and its right child node indicate that attended segments correspond to segments with long-lasting high speed. (High minimum speed in a sliding window, i.e., a high moving minimum of speed, indicates keeping high speed.) Fig. 2g shows a decision tree trained to classify an input time-series into an appropriate class using features extracted from attended segments within the time-series. As shown in the root node and Fig. 2f, the neural network seems to pay attention to the long-lasting high speed of the worms/mice using the attention mechanism, and then distinguishes between DA(+) and DA(−) by the skewness of speed within the attended segments. The attended segments with positive skewness (≥0.360) are classified into the DA(−) class. In contrast, the attended segments with skewness smaller than 0.360 are classified into the DA(+) class (between −0.397 and 0.360). Therefore, these results indicate that the DA(+) worm/mouse keeps stable speed (small skewness) when moving at high speed.

As above, we could extract an interpretable rule from the trained neural network, i.e., DA(−) worms/mice cannot keep high speed. To validate the hypothesis, we perform a statistical test by computing the minimum speed within a time window when the speed is high (see Supplementary Information for details). As shown in Fig. 2h, we observe significant differences between DA(+) and DA(−) for both the mice and worms. Surprisingly, we could also observe significant differences between PD and healthy humans when we performed the same test, even though the neural network was trained only on the data from the mice and worms. See Methods for details about the human data.

As above, our method revealed that humans, mice, and worms with disabilities in the dopaminergic system cannot keep high speed even though their body scales and locomotion methods are completely different. Our study employs worms with a lack of D2 dopamine receptors. Although the PD symptoms are considered to be induced by various impairment of neural circuits such as dopamine transmission impairment, morphological alterations of the basal ganglia circuitry, and lack of dopamine receptors^16–19^, a major source of the discovered locomotion feature shared by PD mice and humans might ascribe the lack of D2 dopamine receptors. The verification of this hypothesis is beyond the scope of this study.

### Network Training on Worm and Human Data

Next, we show results obtained when the neural network is trained on data from worms and humans. As for the human data (see Methods and Supplementary Table 2), we convert time-series of foot-mounted pressure sensors collected during walking into time-series of locomotion speed.

**Fig. 3:**
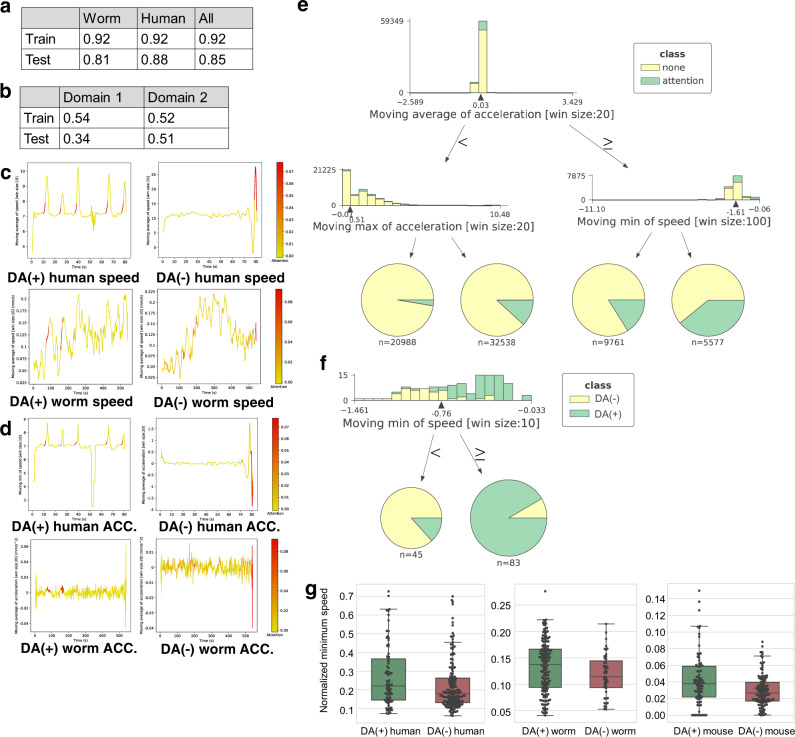
Analysis of DA(+)/DA(−) humans and worms (DA: dopamine). **a** Classification accuracies of our network for DA(+)/DA(−) classes. **b** Classification accuracies of our network for the 1st and 2nd domain estimates; see Supplementary Information for the detailed results of the domain estimates. **c** Example time-series of the speed of DA(+)/DA(−) humans and worms highlighted by our network. **d** Example time-series of acceleration of DA(+)/DA(−) humans and worms highlighted by our network. **e** A decision tree for explaining the meaning of attention. **f** A decision tree for explaining classification. **g** Distributions of minimum speed within a time window when the average acceleration is high for humans and worms (see Supplementary Information for details); significant differences between DA(+) and DA(−) for both humans and worms are observed (worm by the Brunner–Munzel test: *p* = 0.03; *w* = 2.26; df = 101.91; effectsize = 0.60; *n* = 162 from DA(+) worms; *n* = 47 from DA(−) worms; human by the Welch’s *t*-test: *p* = 0.02; *t* = −2.35; df = 126.89; effectsize(r) = −0.34; *n* = 78 from DA(+) humans; *n* = 163 from DA(−) humans); a significant difference between DA(+) and DA(−) for the mice is also observed by the Welch’s *t*-test (*p* = 0, 01; *t* = 2.57; df = 131.63; effectsize(r) = 0.38); *n* = 88 10 min trajectories from DA(+) mice; *n* = 113 10 min trajectories from DA(-) mice; the *p*-value is two sided. The box plot shows the 25–75% quartile, with an embedded bar representing the median and the lower/upper whiskers show Q1-1.5*IQR and Q3+1.5*IQR, respectively, where IQR is the interquartile range, Q1 is 25% quartile, and Q3 is 75% quartile.

We deal with two classes, i.e., DA(+) class (healthy worms and humans) vs. DA(−) class (PD humans and worms lacking D2 dopamine receptors). Fig. 3a, b shows the classification accuracy, indicating that our network seems to extract a cross-species feature between humans and worms. Fig. 3c shows the time-series of speed for DA(+)/DA(−) worms and humans highlighted by the trained model. From these highlighted time-series, the neural network seems to focus on segments corresponding to smooth acceleration for the DA(+) worms and humans. (A decision tree shown below also takes an acceleration feature as a root node.) Fig. 3d shows the time-series of acceleration for worms and humans, as segments of DA(+) worms/humans corresponding to acceleration are attended.

Figure 3e shows a decision tree that is trained to detect the attended segments. The root node and its right child node indicate that attended segments correspond to segments with high acceleration values as well as high minimum speed values within a time window. This result indicates that the neural network focuses on a moment of long-lasting acceleration. Fig. 3f shows a decision tree that is trained to classify an input time-series into an appropriate class using features extracted from attended segments within the time-series. As shown in the root node and Fig. 3e, the neural network seems to pay attention to long-lasting accelerations of the DA(+) worms/humans using the attention mechanism, and then distinguishes between DA(+) and DA(−) simply by the minimum of speed within the attended segments.

From the above results, we can say that the DA(−) worms/humans present unstable speed while accelerating (lower minimum speed during high acceleration). To validate the hypothesis, we perform a statistical test. Based on the decision trees, we compute the minimum speed within a time window when the average acceleration is high (see Supplementary Information for details). As shown in Fig. 3g, we observe significant differences between DA(+) and DA(−) for both humans and worms. Interestingly, we could also observe significant differences between the two classes of mice.

As above, we could notice that the speed of these animals is unstable when accelerating. PD mice used in this study (6-OHDA mouse model of PD) are considered to exhibit motor symptoms of akinesia and bradykinesia^20^. The discovered locomotion feature is similar to the symptom of akinesia. Our study reveals that a similar feature is also found in worms lacking D2 dopamine receptors, indicating that the receptors play an important role in the symptom.

### Network training on worm and beetle data

**Fig. 4:**
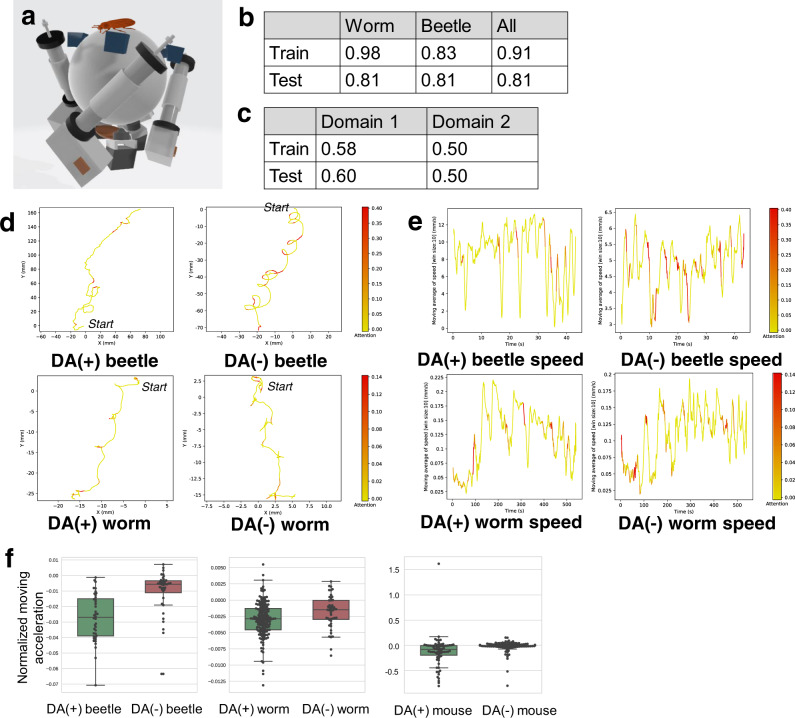
Analysis of DA(+)/DA(−) beetles and worms. (DA: dopamine). **a** Experimental apparatus for beetle study (treadmill). **b** Classification accuracies of our network for DA(+)/DA(−) classes. **c** Classification accuracies of our network for the 1st and 2nd domain estimates; see Supplementary Information for the detailed results of the domain estimates. **d** Example trajectories of DA(+)/DA(−) beetles and worms highlighted by our network. **e** Example time-series of speed of DA(+)/DA(−) beetles and worms highlighted by our network. **f** Distributions of acceleration before turns for beetles and worms (see Supplementary Information for details); significant differences between DA(+) and DA(−) for both beetles and worms are observed (worm by the Brunner–Munzel test: *p* = 0.005; *w* = − 2.90; df = 72.76; effectsize = 0.36; *n* = 162 from DA(+) worms; *n* = 47 from DA(−) worms; beetle by the Brunner–Munzel test: *p* = 2.99 × 10^−8^; *w* = − 6.22; df = 71.50; effectsize = 0.18; *n* = 40 from DA(+) beetles; *n* = 40 from DA(−) beetles); a significant difference between DA(+) and DA(−) for the mice is also observed by the Welch’s t-test (*p* = 0.02; *t* = − 2.37; df = 112.94; effectsize(r) = − 0.35); *n* = 88 10 min trajectories from DA(+) mice; *n* = 113 10 min trajectories from DA(−) mice; the *p*-value is two sided. The box plot shows the 25–75% quartile, with embedded bar representing the median and the lower/upper whiskers show Q1-1.5*IQR and Q3+1.5*IQR, respectively, where IQR is the interquartile range, Q1 is 25% quartile, and Q3 is 75% quartile.

Tonic immobility (sometimes called death-feigning behavior or thanatosis) has been observed in many species and it is thought to have evolved as an anti-predator strategy^21^. The selection regimes for a short or long duration of tonic immobility have been established in the red flour beetle, *Tribolium castaneum*^22^. We use trajectory data of beetles collected from the short and long selection regimes on a treadmill (Fig. 4a; short: 20 beetles; long: 20 beetles). The long selection regime showed significantly lower levels of brain dopamine expression and lower locomotor activity than those of the short selection regime^23^. Further, Uchiyama et al.^24^ showed 518 differentially expressed genes between the selection regimes. As expected from physiological studies described above, genes associated with the metabolic pathways of tyrosine, a precursor of dopamine, were differentially expressed between the short and long selection regime; these enzyme-encoding genes were expressed at higher levels in the long selection regime than in the short selection regime^24^. Therefore, we train the neural network with the long selection regime corresponding to the DA(−) class.

Figure 4b, c show the classification accuracy. Fig. 4d shows trajectories of DA(+)/DA(−) worms and beetles highlighted by the trained model, indicating that the network focuses on segments before the movement direction changes in many cases. Fig. 4e shows the time-series of speed for DA(+)/DA(−) worms and beetles highlighted by the trained model. As shown in these figures, the network focuses on segments before the local minima of speed (corresponding to direction changes, e.g., *t* = 17, 23 for DA(+) beetle). Before the turns, DA(+) worms and beetles seem to quickly decrease their speed. In contrast, DA(−) worms and beetles do not seem to smoothly decrease their speed (e.g., *t* = 15, 20 for DA(−) beetle). Based on the observation, we compare the difference in acceleration before turns between the DA(+) and DA(−) classes (see Supplementary Information for details). As shown in Fig. 4f, we could observe significant differences between DA(+) and DA(−) for both beetles and worms. Yamazaki et al.^15^ revealed that the dopamine-deficient worms are abnormal in speed changes before the initiation of turns, a result which is in keeping with the findings of our study. In addition, recent analysis on DNA sequences of beetles^25^ revealed that DA(−) beetles have large mutations in dopamine-related genes compared with DA(+) beetles, suggesting that DA(−) beetles exhibit abnormal walking behaviors compared with DA(+) beetles due to changes to the dopaminergic system. We could also observe significant differences between the two classes of mice. Because we only have speed time-series for the human data, we could not perform this test for humans.

As above, we observed that DA(−) animals exhibit significantly high acceleration before they change the moving direction. This indicates that the animals with dopamine deficiency cannot smoothly decrease the speed before turns. Specifically, this hypothesis found by our method focuses on the transition from the "running” mode to the "turn” mode. The disability in locomotion mode transition caused by PD has been studied on mice and humans^20,26^. These lines of evidence suggest that the disability is caused by combined factors constituting morphological abnormalities of the neural circuitry and changes of the DA transporter and in the DA receptor densities induced by the lack of DA. On the other hand, our cross-species comparative analysis proposes a hypothesis that the disability can be simply explained by the deficiency of dopamine expression level.

## Discussion

We propose an attention-based domain-adversarial neural network to study cross-domain behavior by analyzing locomotion data from different species. Comparative behavioral analysis between two classes has been performed by using classic classification methods and manual feature design^15,27,28^ as well as studies on locomotion features of PD mice using statistical analysis^1,2^. However, these features are not necessarily observed in other species. In fact, we could not find significant differences between DA(+) and DA(−) for humans (and worms) by calculating the ambulation period, widely used to evaluate PD symptoms of mice^1^ (see Supplementary Figure 1). DeepHL, which is our prior work, is a pioneering study on deep learning-assisted animal behavior analysis using attention mechanisms^12^. However, DeepHL focuses only on behavioral data from a single species and cannot be used to conduct cross-species behavior analysis.

To demonstrate the usefulness of the proposed neural network, we found cross-species locomotion features among species that are far away from each other in the evolutionary lineage. The study reveals that the DA(−) humans, mice, and worms cannot keep high speed. In addition, the speed of the DA(−) humans, mice, and worms is unstable when accelerating. Moreover, the DA(−) worms, mice, and beetles present significantly high acceleration before they changing direction. We believe that discovering such hypotheses from time-series trajectory data obtained from multiple species is difficult. In this study, we focused on specific important moments in the time-series highlighted by the attention mechanisms, e.g., moments of acceleration and moments before the turn. However, it is difficult to focus on such moments by manually analyzing trajectories from multiple species.

While training the neural network using data from two species, the discovered locomotion features from one of them were observed in other species as well, indicating that the neural network captures latent locomotion features across different species. As a result, we propose the hypothesis that various aspects of motor dysfunction across animal species can be explained by the deficiency of dopamine expression levels. Our method could thus be useful in identifying animal models for a variety of Parkinson’s disease symptoms such as akinesia, bradykinesia, tremor, rigidity, and postural instability.

In practice, researchers may label trajectories mistakenly. For example, some DA(+) trajectories can be mistakenly labeled as DA(−). Our additional investigation on the robustness of our method against potential labeling errors revealed that, even when 5% of erroneous labels are included, our model maintains high classification accuracy. See Supplementary Information and Supplementary Figure 4 for more detail.

Here we discuss the rationale for using a decision tree in the process of explaining the meaning of attended segments. The aim of this process is to help understand the meaning of the attended segments. It is therefore important to explain the meaning of attention using a small number of features. Even though regression models enable us to detect important features highly correlated with the attended segments, the regression models suffer from multicollinearity. Because some locomotion features–such as speed and acceleration–are correlated with each other, it is difficult to use the regression models in this procedure. Although the support vector machine (SVM) is a widely-used classifier and achieves high classification accuracy, the interpretability of the model is poor because the SVM learns a classification rule in a high-dimensional feature space when many features are available. Another possible approach is to use a method for evaluating locomotion features. This approach provides us with features useful in detecting attended segments. However, unlike decision trees, this approach does not provide information about thresholds, making it difficult to understand the meaning of the attended segments (see Supplementary Information). In contrast, a decision tree describes classification rules using a hierarchy of if-then rules with thresholds, enabling us to easily understand the meaning of the attended segments based on a small number of features existing in shallower nodes in the tree. We believe that the findings by our method are intuitive and can be easily understandable by non-computer scientists.

Domain-adversarial neural networks have been originally proposed for transfer learning^8^. To the best of our knowledge, this is the first study that employs them for highlighting cross-species behavioral features. Moreover, this study introduces the attention mechanism to the domain-adversarial network in order to interpret the discovered cross-species behavioral features by the neural network.

The proposed method can be potentially applied to evaluate animal models of other diseases, accelerating therapeutic drug development. Our approach enables the formation of interpretable rules for evaluation, preventing the evaluation of diseases using a black-box model. In addition, we believe that our method can be applied to human society and industrial domains. For example, our method enables the extraction of inherent locomotion features of high-performing taxi drivers across different cities. Moreover, our method enables the extraction of inherent locomotion features of high-performing workers performing order-picking in different logistics centers. Because these features are expected to be used to train low-performing drivers/workers, the interpretability of the features is important.

This study employs trajectory data from two species to train a neural network, and investigates whether a found cross-species feature constitutes a significant difference in another species. However, when, for example, we have trajectory data from 100 species, it is difficult to discover locomotion features shared across the 100 species from a network trained on data from only two species. We therefore believe that improving our method so that it can deal with more than two species will be important in future work.

A potential limitation of our method is the trade-off between the interpretability and statistical significance of the finding (a rule obtained from our method). In our experiment, we obtained interpretable rules regarding highlighted segments based on shallow nodes in decision trees. In general, a rule with high interpretability can yield low statistical significance. If we can automatically make a rule while controlling its interpretability, such as by using tree nodes including deeper levels, we can also control the statistical significance of the rule. We believe that developing a technology that enables the control of interpretability depending on a user’s needs can be one of the most important directions toward explainable artificial intelligence.

## Methods

### Worm data

Young adult wild-type hermaphrodite *Caenorhabditis elegans* (*C. elegans*) were cultivated with the bacteria OP50 and handled as described previously^29^. The *C. elegans* wild-type Bristol strain (N2) were obtained from the Caenorhabditis Genetics Center (University of Minnesota, USA), and *dop-3(tm1356)* was obtained from National Bioresource Project (Japan) and backcrossed with N2 two times to generate KDK1.

The behavioral trajectories of wild-type ("normal”) and *dop-3* worms were monitored during avoidance behavior to the repulsive odor 2-nonanone (normal: 162; dop-3: 47)^30^. During the odor avoidance, naive wild-type and *dop-3* worms exhibit essentially similar behavioral responses although they behave differently after pre-exposure to the odor, indicating that their naive response to the odor is not dependent of dopamine signaling, while their response after pre-exposure is^30^. Thus, in this study, we focused on the behavior of wild-type and *dop-3* worms after pre-exposure to the odor. The odor avoidance behavior was monitored with a high-resolution fixed USB camera for 12 min at 1 Hz. Because the worms do not exhibit odor avoidance behavior during the first 2 min because of the rapid increase in odor concentration^31^, only the data from the following 10 min (i.e., 600 s) was used^14^. We employed Move-tr/2D (Library Inc., Japan; v. 8.31) to track to automatically track the worms’ centroids in the camera images. A part of the original data had already been analyzed and published^14,15^, being re-analyzed in this study.

We trimmed /undersampled the trajectory data in order to make data lengths of all trajectories (of humans, mice, and beetles) identical before feeding them into the neural network. For more details about the trajectory data of the four animals, see Supplementary Table 2.

### Mouse data

Nine C57BL/6J mice purchased from Shimizu Laboratory Supplies (Kyoto, Japan) (male or female; 6–17 months old at the beginning of the experiment) were housed in groups at 23 ^∘^C and humidity of 40–60%, with food and water provided ad libitum in a 12 h light and 12 h dark cycle (day starting at 09:00). All tests were performed during the light period. For PD mice, under isoflurane anesthesia, 6-OHDA (4 mg/ml; Sigma) was injected through the implanted cannulae (AP -1.2 mm, ML 1.1 mm, DV 5.0 mm, 2 μl). The PD mice were allowed to recover for at least one week before post-lesion behavioral testing. After the mice were sacrificed by pentobarbital sodium overdose and perfused with formalin, their brains were frozen and cut coronally at 30 μl with a sliding microtome. For immunostaining, sections were divided into six interleaved sets. Immunohistochemistry was performed on the free-floating sections. Sections were pretreated with 3% hydrogen peroxide and incubated overnight with primary antibody mouse anti-tyrosine hydroxylase (1:1000; Millipore). As a secondary antibody, we used biotinylated donkey anti-mouse IgG (1:100; Jackson ImmunoResearch Inc.) followed by incubation with avidin-biotin-peroxydase complex solution (1:100; VECTASTAIN Elite ABC STANDARD KIT, Vector laboratories). To estimate the degree of dopaminergic cell loss, we divided the number of cells manually counted across three sections of the SNc (most rostral, most caudal, and the intermediate between them) of the lesioned hemisphere from that of the non-lesioned hemisphere.

We collected 52 trajectories of five normal mice and four unilateral 6-OHDA lesioned mouse models of PD while they freely walked for 10 min in an open arena (60 × 55 cm, wall height = 20 cm; normal: 22, PD: 30). The trajectories were tracked from the animal’s head position extracted from images captured by a digital video camera (60 fps) mounted on the ceiling of the enclosure. We used custom software based on Matlab (R2018b, Mathworks, Ma, USA) and LabVIEW (Labview 2018, National Instruments, TX, USA) to track the mice. Two sets of small red and green light-emitting diodes mounted above the animal’s head were used to track its location in each frame. We then created 150 s segments by splitting each trajectory because training a neural network requires a number of trajectories. We used 201 segments in total (normal: 88, PD: 113) collected from the mice. Note that we excluded 150 s segments that contain no movements of a mouse.

### Human data

We employed a publicly available gait data set of normal and Parkinson’s disease humans (Gait in Parkinson’s Disease Dataset)^32^. In brief, this data set contains measures of gait from 93 patients with idiopathic PD and 73 healthy controls. The data set includes the vertical ground reaction force records from force 8 sensors underneath each foot with the 100 Hz sampling rate as the subjects walked at their usual for approximately 2 min. Note that our study did not use data collected during dual-tasking (serial 7 subtractions). Because the duration of almost all of the data were 82 s, we also did not use data shorter than 82 s.

### Beetle data

We analyzed 80 walking trails of beetles collected from short and long selection regimes strain beetles on a treadmill system^33^. We used custom software based on OpenCV (https://opencv.org/; v. 2.4.9) for tracking the beetles. The stock population of *Tribolium castaneum* used in the present study has been maintained in laboratories for more than 25 years. The beetles are fed a blend of whole wheat flour and brewer’s yeast at a 19:1 ratio. They are kept in an incubator (Sanyo, Osaka, Japan) maintained at 25 ^∘^C under a 16 h light:8 h dark cycle. The selection regimes with short and long duration of tonic immobility were used^34^. The number of beetles derived from the short selection regime (long selection regime) is 20, consisting of 10 males and 10 females.

### Preprocessing of trajectories

We first convert a trajectory into a time-series of speed. Let *P* be an input trajectory that consists of a sequence of two-dimensional positions with timestamps:

1 $$P	= \;\; [{P}_{1},{P}_{2},...,{P}_{T}]\\ 	= \;\; [({t}_{1},{x}_{1},{y}_{1}),({t}_{2},{x}_{2},{y}_{2}),...,({t}_{T},{x}_{T},{y}_{T})]$$

For the trajectories of animals whose absolute coordinates are meaningless, such as those of animals that freely move on an agar plate, the relative position analysis is required. Therefore, we convert *P* into *S*, which is a sequence of speeds:

2 $$S=[{s}_{2},{s}_{3},...,{s}_{T}]$$

where *s*_*i*_ is the speed at the time *i* and described as

3 $${s}_{i}=\frac{\,{{\mbox{Dist}}}\,({P}_{i},{P}_{i-1})}{{t}_{i}-{t}_{i-1}},$$

where Dist(. , . ) computes the Euclidean distance between two coordinates. After this, we normalize the speed time-series for each trajectory. Each speed time-series is associated with a class label and domain label.

### Processing of human gait data

Because the stride time (i.e., the time elapsed between the first contact of two consecutive footsteps of the same foot) is proportionate to the walking speed^35^, we first compute time-series of stride time for each foot. We then combine the two time-series, i.e., by sorting data points of stride times from the two time-series by their time stamps. After that, we standardize a set of speed time-series. Each speed time-series is associated with a class label and domain label.

### Attention-based domain-adversarial deep neural network

Here, we explain the proposed deep neural network model shown in Fig. 1d in detail. The input of the model is a speed time-series *S* with the length of *l*. In each 1D convolutional layer of the feature extraction block, we extract features by convolving the input time-series through the time dimension using a filter with a width of *F*_*t*_. We use a stride (step size) of 1 sample in terms of the time axis. In addition, to reduce overfitting, we employ dropout, which is a simple regularization technique in which randomly selected neurons are dropped during training^36^. The dropout rate used in this study is 0.5.

We also employ 1D convolutional layers in the attention computation block to compute attention time-series from the input time-series *S*. The attention layer in the attention computation block computes attention from an output matrix of the 2nd convolutional layer *Z* as follows.

4 $${{{{{{{\bf{a}}}}}}}}={{{{{{{\rm{softmax}}}}}}}}({W}_{a2}\cdot \tanh ({W}_{a1}{Z}^{T})),$$

where **a** is an attention vector of length *l* that shows the importance (i.e., attention) of each data point in the input time-series. Because the attention layer is implemented as two densely connected layers with no bias, *W*_*a*1_ and *W*_*a*2_ show the weight matrices of the 1st and 2nd densely connected layers, respectively. The softmax function ensures that the output values sum to 1, and the $\tanh$ function limits the output value of its input to a value between -1 and 1. The attention is multiplied by the outputs of the 2nd convolutional layer in the feature extraction block to contrast the segments to which the network pays attention.

The extracted features multiplied by the attention are fed into the class predictor and the 1st domain predictor, which are composed of two densely connected layers. The 1st densely connected layers employ the $\tanh$ activation function. The 2nd layers (output layers) employ the softmax function. The class predictor and 1st domain predictor output class and domain estimates, respectively. With the gradient reversal layer in the 1st domain predictor, we make the network incapable of estimating domains from extracted features, which is described in detail later.

The output of the 2nd convolutional layer of the attention computation block is fed into the 2nd domain predictor for each time step. The 2nd domain predictor also consists of two densely connected layers. The 1st densely connected layer employs the $\tanh$ activation function. The 2nd layer (output layer) employs the softmax function. The 2nd domain predictor outputs a domain estimate for each time step. With the gradient reversal layer in the 2nd domain predictor, we make the way of attention computation domain-independent.

### Network training

We train the neural network using the backpropagation algorithm^11^. The gradient reversal layer in the network multiplies the gradient with a negative constant value (−μ) when we train the network using the backpropagation algorithm. Because the parameters in the feature extraction and attention computation blocks are updated so that the domain estimates become worse, the gradient reversal layer makes the neural network incapable of distinguishing between the two domains. Note that achieving these different goals at the same time makes it difficult for the neural network to converge. Therefore, we iterate the three procedures described in the main text to train the network. Here we explain the procedures in detail.

The first procedure minimizes the error of the class estimates as well as maximizes the error of the domain estimates by the 2nd domain predictor by minimizing

5 $$E({\theta }_{f},{\theta }_{a},{\theta }_{c},{\theta }_{d2})=\frac{1}{n}\mathop{\sum }\limits_{i=1}^{n}{{{{{{{{\mathcal{L}}}}}}}}}_{c}^{i}({\theta }_{f},{\theta }_{a},{\theta }_{c})-{\lambda }_{1}\frac{1}{n}\mathop{\sum }\limits_{i=1}^{n}{{{{{{{{\mathcal{L}}}}}}}}}_{d2}^{i}({\theta }_{a},{\theta }_{d2}),$$

where *θ*_*f*_, *θ*_*a*_, *θ*_*c*_, *θ*_*d*2_ represent network parameters of the feature extraction block, attention computation block, class predictor, and 2nd domain predictor, respectively, *n* is the number of training instances (time-series), ${{{{{{{{\mathcal{L}}}}}}}}}_{c}^{i}({\theta }_{f},{\theta }_{a},{\theta }_{c})$ shows the loss of class prediction, and ${{{{{{{{\mathcal{L}}}}}}}}}_{d2}^{i}({\theta }_{a},{\theta }_{d2})$ is the loss of domain prediction by the 2nd domain predictor. The hyperparameter *λ*_1_ is used to trade-off between the two-loss functions. ${{{{{{{{\mathcal{L}}}}}}}}}_{c}^{i}({\theta }_{f},{\theta }_{a},{\theta }_{c})$ in Equation (5) shows the binary cross-entropy loss for class estimates described as follows

6 $${{{{{{{{\mathcal{L}}}}}}}}}_{c}^{i}({\theta }_{f},{\theta }_{a},{\theta }_{c})=-({y}_{i}\cdot {{{{{{\mathrm{log}}}}}}}\,{p}^{C}({S}_{i}| {\theta }_{f},{\theta }_{a},{\theta }_{c})+(1-{y}_{i})\cdot {{{{{{\mathrm{log}}}}}}}\,(1-{p}^{C}({S}_{i}| {\theta }_{f},{\theta }_{a},{\theta }_{c}))),$$

where *y*_*i*_ is a ground truth class label (0 or 1) of the *i*-th time-series *S*_*i*_ and *p*^*C*^(*S*_*i*_∣*θ*_*f*_, *θ*_*a*_, *θ*_*c*_) is a class estimate by the class predictor. ${{{{{{{{\mathcal{L}}}}}}}}}_{d2}^{i}({\theta }_{a},{\theta }_{d2})$ shows the binary cross-entropy loss for attention for each time step described as follows

7 $${{{{{{{{\mathcal{L}}}}}}}}}_{d2}^{i}({\theta }_{a},{\theta }_{d2})\!=\!-\frac{1}{T-1}\mathop{\sum }\limits_{t=2}^{T}{d}_{i}\cdot {{{{{{\mathrm{log}}}}}}}\,{p}^{D}({s}_{i,t}| {\theta }_{a},{\theta }_{d2})\!+\!(1\!-\!{d}_{i})\cdot {{{{{{\mathrm{log}}}}}}}\,(1\!-\!{p}^{D}({s}_{i,t}| {\theta }_{a},{\theta }_{d2})),$$

where *d*_*i*_ is a ground truth domain label (0 or 1) of the *i*-th time-series, *s*_*i*,*t*_ is a data point in the *i*-th time-series at time *t* and *p*^*D*^(*s*_*i*,*t*_∣*θ*_*a*_, *θ*_*d*2_) is a domain estimate by the 2nd domain predictor.

The second procedure maximizes the error of the domain estimates by the 1st domain predictor by minimizing

8 $$E({\theta }_{f},{\theta }_{a},{\theta }_{d1})=\frac{1}{n}\mathop{\sum }\limits_{i=1}^{n}{{{{{{{{\mathcal{L}}}}}}}}}_{d1}^{i}({\theta }_{f},{\theta }_{a},{\theta }_{d1}),$$

where *θ*_*d*1_ is the network parameter of the 1st domain predictor and ${{{{{{{{\mathcal{L}}}}}}}}}_{d1}^{i}({\theta }_{f},{\theta }_{a},{\theta }_{d1})$ is the loss of domain prediction by the 1st domain predictor. ${{{{{{{{\mathcal{L}}}}}}}}}_{d1}^{i}({\theta }_{f},{\theta }_{a},{\theta }_{d1})$ in Equation (8) shows the binary cross-entropy loss for domain estimates described as follows

9 $${{{{{{{{\mathcal{L}}}}}}}}}_{d1}^{i}({\theta }_{f},{\theta }_{a},{\theta }_{d1})=-({d}_{i}\cdot {{{{{{\mathrm{log}}}}}}}\,{p}^{D}({S}_{i}| {\theta }_{f},{\theta }_{a},{\theta }_{d1})+(1-{d}_{i})\cdot {{{{{{\mathrm{log}}}}}}}\,(1-{p}^{D}({S}_{i}| {\theta }_{f},{\theta }_{a},{\theta }_{d1}))),$$

where *p*^*D*^(*S*_*i*_∣*θ*_*f*_, *θ*_*a*_, *θ*_*d*1_) is a domain estimate by the 1st domain predictor.

The third procedure consists in minimizing

10 $$E({\theta }_{f},{\theta }_{a},{\theta }_{c},{\theta }_{d1})=\frac{1}{n}\mathop{\sum }\limits_{i=1}^{n}{{{{{{{{\mathcal{L}}}}}}}}}_{c}^{i}({\theta }_{f},{\theta }_{a},{\theta }_{c})-{\lambda }_{2}\frac{1}{n}\mathop{\sum }\limits_{i=1}^{n}{{{{{{{{\mathcal{L}}}}}}}}}_{d1}^{i}({\theta }_{f},{\theta }_{a},{\theta }_{d1}),$$

where *λ*_2_ is a trade-off hyperparameter of the two-loss functions.

We employ the algorithm Adam^37^ in each procedure to minimize the loss functions. Note that, because parameters in the network are unstable in the earlier epochs, using large *μ* makes it difficult for the network to converge. Therefore, in the earlier epochs, we use small *μ* to properly train the feature extraction block and then gradually increase *μ* as follows.

11 $$\mu =\left\{\begin{array}{ll}0\hfill&(0\le i\, < \, {T}_{1})\hfill\\ \frac{L+1}{L\cdot ({\alpha }^{-\beta \frac{i-{T}_{1}}{{T}_{2}-{T}_{1}}})+1}-1&({T}_{1}\le i \, < \, {T}_{2})\\ L \hfill&({T}_{2}\le i)\hfill\end{array}\right.$$

where *i* is the epoch number, *L* is the upper bound of *μ*, *α* = 1.4, and *β* = 10. *T*_1_ and *T*_2_ are empirically determined as ${T}_{1}=\frac{1}{10}\cdot \#epochs$ and ${T}_{2}=\frac{1}{1.2}\cdot \#epochs$, where *#epochs* indicate the number of training epochs.

### Decision tree for explaining attention

We build a decision tree that explains the meaning of attention by the network using attention outputs by the network as training labels. We first extract a feature vector from the input time-series for each time slice. We extract interpretable features for each data point described in Supplementary Table 1. We then label the feature vectors according to attention outputs by the network. When an attention value at time *t* is higher than a given threshold, we label a feature vector at time *t* as "attended.” Otherwise, we label as "none.” Note that, because the softmax function in the attention computation block ensures that all attention values in an input time-series sum to 1, we set the threshold as $\frac{1}{T-1}$. Then we train a binary classifier using the labeled feature vectors. With the trained decision tree, the user can understand the meaning of the extracted attention.

### Decision tree for explaining classification

We build a decision tree that explains the meaning of classification by the attention-based neural network. We first construct a feature vector for each input time-series by averaging a feature vector prepared for each sliding time window, which is extracted in the same way as that of training a decision tree for explaining attention. Note that, when averaging, we calculate the weighted average according to the attention value by the network. For example, when an attention value of an input time-series at time *t* is *a*_*t*_, we multiply *a*_*t*_ to a feature vector at time *t*. By doing so, we can build a rule mainly focusing on attended segments. After that, we train a decision tree using the averaged feature vectors with their class labels (e.g., DA(+) or DA(−) class). With the trained decision tree, the user can understand the meaning of the classification by taking into account attention by the network.

### Ethics statement

The study on mice was approved by the Doshisha University Institutional Animal Care and Use Committees.

### Reporting summary

Further information on research design is available in the Nature Research Reporting Summary linked to this article.

## Supplementary information

Supplementary Information

Description of Additional Supplementary Files

Supplementary Software 1

Reporting Summary

## Data Availability

The data of mice, beetles, and worms are available with Supplementary Software 1 and 10.5281/zenodo.5142294^38^. Source data are provided with this paper.

## Source data

Source Data

## Code availability

The software used in this study are available as Supplementary Software 1. The most recent version of the software is available at 10.5281/zenodo.5142294^38^. The software was implemented based on Python (v.3.6.0), numpy (v.1.15.2), scikit-learn (v.0.20.0), scipy (v.1.2.1), and tensorflow (v.1.12.0).
