# Supplementary material for: Cross-species behavior analysis with attention-based domain-adversarial deep neural networks
Source: Nat Commun. 2021 Sep 17;12:5519. doi: 10.1038/s41467-021-25636-x (PMC8448872; doi:10.1038/s41467-021-25636-x)
Supplement: Supplementary file 1 — Supplementary Information [file 41467_2021_25636_MOESM1_ESM.pdf]

Supplementary Notes for

Cross-species Behavior Analysis with Attention-based Domain-adversarial Deep Neural Networks

Takuya Maekawa, Daiki Higashide, Takahiro Hara, Kentarou Matsumura, Kaoru Ide, Takahisa Miyatake, Koutarou D. Kimura, & Susumu Takahashi

Takuya Maekawa.
E-mail: maekawa@ist.osaka-u.ac.jp

Contents

Training on Data from Worms and Mice 2

Training on Data from Worms and Humans 2

Training on Data from Worms and Beetles 2

Investigation of the Robustness of the Method against Labeling Errors 3

Analysis of features for detecting attended segments using ANOVA 3

User Guide 3

Training on Data from Worms and Mice

We randomly split the trajectory data with a ratio of 80 percent for training and 20 percent for testing, and then train our neural network on the training data with 30,000 epochs. The decision tree for explaining the meaning of attention was trained on feature vectors from the training data. The test classification accuracy of the decision tree for explaining the meaning of attention was 0.86. The test classification accuracy of the decision tree for explaining the classification was 0.81. Supplementary Figure 5a shows the classification accuracies of our network for the 1st and 2nd domain estimates for each domain and class. As shown in the results, the accuracies of the 1st domain estimates for the worms are 1.0. In contrast, the accuracies of the 1st domain estimates for the mice are about 0.0. This result means that the 1st domain predictor classified almost all the trajectories into the worm domain, indicating that the domain predictor could not identify domain-specific features. The accuracies for the 2nd domain predictor were also imbalanced as shown in Supplementary Figure 5a. The 2nd domain predictor also seems not to be able to identify domain-specific features in the outputs of the attention computation block.

For the statistical test, we computed the rolling minimum of the normalized speed within segments with high speed (top 20% average speed) for each trajectory. The average speed was computed for each 20-sample sliding window. These feature parameters were determined based on thresholds of important features used in the decision trees. In addition to the mice and worms, we could observe significant difference for the humans by the Welch's t-test ($p = 0.03$; $t = 2.25$; df $= 123.83$; effect size(r) $= -0.32$) as shown in Supplementary Figure 2a. In contrast, we could not observe significant difference for the beetles by the Brunner-Munzel test ($p = 0.92$; $w = -0.10$; df $= 77.81$; effect size $= 0.49$). For the worms, mice, and humans, the DA(+) individuals keep stable speed (higher minimum speed during high speed movement) than the DA(-) individuals.

Here we introduce the results of the normality tests (Shapiro-Wilk's test) of the distributions of the animals related to the proposed feature. The distributions of the DA(+) and DA(-) classes for the mice were normally distributed (DA(+): $p = 0.003$; $W = 0.95$; df $= 88$; DA(-): $p = 3.24 \times 10^{-5}$; $W = 0.93$; df $= 113$). The distribution of the DA(+) class for the worms was normally distributed ($p = 0.002$; $W = 0.97$; df $= 162$). The distribution of the DA(-) class for the worms was not normally distributed ($p = 0.47$; $W = 0.98$; df $= 47$). The distributions of the DA(+) and DA(-) classes for the humans were normally distributed (DA(+): $p = 1.25 \times 10^{-5}$; $W = 0.90$; df $= 78$; DA(-): $p = 2.73 \times 10^{-12}$; $W = 0.83$; df $= 163$). The distributions of the DA(+) and DA(-) classes for the beetles were not normally distributed (DA(+): $p = 0.06$; $W = 0.95$; df $= 40$; DA(-): $p = 0.06$; $W = 0.94$; df $= 40$).

Training on Data from Worms and Humans

We train the network using the same way as that of the study on worms and mice. The test classification accuracy of the decision tree for explaining the meaning of attention was 0.94. The test classification accuracy of the decision tree for explaining the classification was 0.67. Supplementary Figure 5b shows the classification accuracies of our network for the 1st and 2nd domain estimates for each domain and class. As shown in the results, the 1st domain predictor mistakenly classified the worm trajectories belonging to DA(+). In addition, the 1st domain predictor mistakenly classified the human trajectories belonging to DA(-). These results indicate that, although features useful to distinguish between DA(+) and DA(-) are preserved, features enabling to distinguish between the domains (i.e., worm vs. human) are eliminated.

For the statistical test, we computed the rolling minimum of the normalized speed with high acceleration (top 10% average acceleration) for each trajectory. The average acceleration was computed for each 20-sample sliding window. In addition to the humans and worms, we could observe significant differences for the mice by the Welch's t-test ($p = 0,01$; $t = 2.57$; df $= 131.63$; effect size(r) $= 0.38$). However, we could not observe significant difference for the beetles by the Brunner-Munzel test ($p = 0.05$; $w = -1.96$; df $= 64.25$; effect size $= 0.37$) as shown in Supplementary Figure 2b. For the worms, mice, and humans, the DA(-) individuals cannot accelerate long time (lower minimum speed during high acceleration) than the DA(+) individuals.

Here we introduce the results of the normality tests (Shapiro-Wilk's test) of the distributions of the animals related to the proposed feature. The distributions of the DA(+) and DA(-) classes for the humans were normally distributed (DA(+): $p = 1.20 \times 10^{-5}$; $W = 0.90$; df $= 78$; DA(-): $p = 2.00 \times 10^{-12}$; $W = 0.83$; df $= 163$). The distribution of the DA(+) class for the worms was normally distributed ($p = 0.01$; $W = 0.98$; df $= 162$). The distribution of the DA(-) class for the worms was not normally distributed ($p = 0.47$; $W = 0.98$; df $= 47$). The distributions of the DA(+) and DA(-) classes for the mice were normally distributed (DA(+): $p = 1.16 \times 10^{-5}$; $W = 0.91$; df $= 88$; DA(-): $p = 4.99 \times 10^{-5}$; $W = 0.93$; df $= 113$). The distribution of the DA(+) and DA(-) classes for the beetles were not normally distributed (DA(+): $p = 0.18$; $W = 0.96$; df $= 40$; DA(-): $p = 0.16$; $W = 0.96$; df $= 40$).

Training on Data from Worms and Beetles

Supplementary Figure 5c shows the classification accuracies of our network for the 1st and 2nd domain estimates for each domain and class. Similar to the results of the investigation of thw worms and humans, the 1st domain predictor mistakenly classified the worm trajectories belonging to DA(+). In addition, the 1st domain predictor mistakenly classified the beetle trajectories belonging to DA(-). These results also indicate that features enabling to distinguish between the domains (i.e., worm vs. beetle) are eliminated.

For the statistical test, we computed the normalized moving average of acceleration just before curves (three-time steps before curve). The curve was defined as the local minimum of the moving averaged angular speed. The moving average of

acceleration and angular speed were computed for each 10-sample sliding window. In addition to the beetles and worms, we could observe significant differences for the mice by the Welch's t-test ($p = 0.02$; $t = -2.37$; df $= 112.94$; effect size(r) $= -0.35$) as shown in Supplementary Figure 2c. The above hypothesis was obtained by watching highlighted trajectories and time-series of speed as shown in Fig. 4d and e.

Here we introduce the results of the normality tests (Shapiro-Wilk's test) of the distributions of the animals related to the proposed feature. The distribution of the DA(+) class for the beetles was not normally distributed ($p = 0.32$; $W = 0.97$; df $= 40$). The distribution of the DA(-) class for the beetles was normally distributed ($p = 6.37 \times 10^{-10}$; $W = 0.74$; df $= 40$). The distribution of the DA(+) class for the worms was normally distributed ($p = 0.003$; $W = 0.97$; df $= 162$). The distribution of the DA(-) class for the worms was not normally distributed ($p = 0.37$; $W = 0.97$; df $= 47$). The distributions of the DA(+) and DA(-) classes for the mice were normally distributed (DA(+): $p = 5.10 \times 10^{-12}$; $W = 0.70$; df $= 88$; DA(-): $p = 2.17 \times 10^{-15}$; $W = 0.57$; df $= 97$).

Supplementary Figure 3a shows a decision tree that is trained to detect the attended segments. The test classification accuracy of the decision tree for explaining the meaning of attention was 0.62. In addition, Supplementary Figure 3b shows a decision tree that is trained to classify an input time-series into an appropriate class using features extracted from attended segments within the time-series. The test classification accuracy of the decision tree for explaining the classification was 0.75. Here, we derive a hypothesis related to cross-species locomotion features using the decision trees. As shown in Supplementary Figure 3a, attended segments correspond to segments with small speed values as well as high minimum speed values within a time window. This result indicates that the neural network focuses on a moment of slow speed. As shown in Supplementary Figure 3b, the neural network seems to pay attention to slow speed segments using the attention mechanism, and then detects trajectories belonging to the DA(+) class by the negative skewness of speed within the attended segments. The negative skewness of speed means that DA(+) worms/beetles suddenly decelerate when moving slowly. To validate the hypothesis, we perform a statistical test. Based on the hypothesis, we compute the rolling minimum of normalized speed within a time window when the speed is slow (low 5% moving averaged speed). However, we could not observe significant difference between DA(+) and DA(-) only for the worms by the Welch's t-test (worm: $p = 0.09$; $t = -1.74$; df $= 58.71$; effect size(r) $= 0.32$; beetle: $p = 0.04$; $t = 2.10$; df $= 47.23$; effect size(r) $= 0.48$) as shown in Supplementary Figure 3c. The p-value is two sided.

Investigation of the Robustness of the Method against Labeling Errors

In practice, researchers may label trajectories mistakenly. To investigate the robustness of our method against the labeling errors, we trained the proposed neural network by randomly introducing erroneous class labels of training trajectories. Supplementary Figure 4 shows the transition of the classification accuracies when we varied the ratio of training trajectories with erroneous labels in the investigation of the worms and mice. Supplementary Figure 4 also shows the transitions of the classification accuracies of our network for the 1st and 2nd domain estimates. As shown in the result, even when 5% of erroneous labels are included, our model maintains 75% classification accuracy. However, when the ratio of the erroneous labels is larger than 5%, the classification accuracy gradually decreases.

Analysis of features for detecting attended segments using ANOVA

Our method employs decision trees to help understand the meaning of attended segments. Here we investigate another way to understand the meaning of attended segments. We evaluate features listed in Table 1 in the study of the worms and mice using ANOVA in terms of attended segment detection. Supplementary Table 3 shows the top-five important features in terms of F-values. Similar to the results of decision tree analysis (Figure 2f), we can see that the moving average of speed and moving minimum of speed are important. However, it is difficult to understand the meaning of the attended segments using just enumerating the important features. We believe that decision trees, which provide information regarding the distributions of important features for attended segments, are suitable for this analysis.

User Guide

We introduce the Python-based software, which runs on an Ubuntu computer with a GPU board (Supplementary Software). We tested the operation of our software on a desktop computer with the following specifications: Intel Core i5-9600K CPU, 32 GB memory, Nvidia GeForce GTX Titan RTX GPU, and the Ubuntu 18.04 LTS operating system. In addition, the following software was installed: Anaconda v4.5.11, CUDA v9.0.0, CuDNN v7.3.1, Python v3.6.8, Tensorflow (gpu): v1.8.0. The other libraries required for the software are described in the readme document. It takes about half hour to install the above software. Our software contains the following main files:

- twodomain_train.py: for training a neural network on training data.
- get_attention.py: for outputting intermediate outputs of the network, i.e., attention.
- decision_tree_check.py: for training two types of decision trees.
- plot_highlight.py: for outputting highlighted trajectories and time-series.
- show_images.py: for outputting HTML files that enumerate images of highlighted trajectories and time-series.

Because our programs are written in Python, it takes no time to install the software. Here, we explain how the user prepares trajectory data files. The user prepares two types of data: trajectory and normalized features. For trajectory data, the user first prepares a data folder "trajectory" containing the data. The "trajectory" contains four data folders (e.g., da_plus_worm, da_minus_worm, da_plus_mouse, da_minus_mouse). Each folder stores a CSV file prepared for each trajectory. We explain the format of a CSV file containing a trajectory. The first column of the file corresponds to the timestamp (numeric), the second column corresponds to the angular speed, the third column corresponds to the speed, the forth column corresponds to the X coordinate, and the fifth column corresponds to the Y coordinate. (Our software does not use angular speed information.) The first line of the file is the header. For normalized feature data, the user prepares a data folder "normalized_dataset/normalize_each/dataset" containing the data. The "dataset" folder contains four data folders (e.g., da_plus_worm, da_minus_worm, da_plus_mouse, da_minus_mouse). (Note that the normalization method can be implemented by each user. This study employs min-max normalization for each trajectory.) Each folder stores a CSV file prepared for each trajectory. The first column of the file corresponds to the normalized angular speed and the second column corresponds to the normalized speed. The first line of the file is the header. In addition, the user prepares a data folder "normalized_dataset/normalize_each/trajectory" containing four data folders. Each folder stores a CSV file for each trajectory. The first column of the file corresponds to the timestamp (numeric), the second column corresponds to the normalized angular speed, the third column corresponds to the normalized speed, the forth column corresponds to the X coordinate, and the fifth column corresponds to the Y coordinate.

Here, we present the commands for running our software using an example data set associated with the software. It takes about three hours to complete the demo. Because our algorithm relies on deep learning, the reproducibility is not guaranteed.

1. Training neural network

```
python twodomain_train.py '1' '0.2' 'normalized_dataset/normalize_each/dataset'
       'worm' 'trans-norm' 'trans-dop3' 'mouse_sm2' 'normal-pre_sm2' 'pd-pre_sm2'
```

'1' shows the number of epochs (iterations) that use the same condition (specify '1'). '0.2' shows the upper bound of μ (parameter of gradient reversal). 'normalized_dataset/normalize_each/dataset' stores normalized feature data. 'worm' is the 1st domain name. 'trans-norm' is a name of the 1st class of the 1st domain (corresponding to folder name in 'normalized_dataset/normalize_each/dataset'). 'trans-dop3' is a name of the 2nd class of the 1st domain. 'mouse_sm2' is the 1st domain name. 'normal-pre_sm2' is a name of the 1st class of the 2nd domain. 'pd-pre_sm2' is a name of the 2nd class of the 2nd domain. This command stores the trained model in a child directory of the 'model' directory. The name of the child directory shows the name of the training session, which is used in the following commands. This command also stores the transitions of accuracies as a CSV file in the 'accuracy' directory. The first column of the file shows the epoch number. 'xxx:Cacc' column shows the trajectory classification accuracy for xxx class calculated using training data. 'xxx:CaccT' column shows the trajectory classification accuracy for xxx class calculated using test data. 'Dacc(at)' column shows the 2nd domain classification accuracy (attention) calculated using training data. 'Dacc(xm)' column shows the 1st domain classification accuracy (trajectory) calculated using training data. 'DaccT(at)' and 'DaccT(xm)' columns show the domain classification accuracies using test data.

2. Outputting attention values

```
python get_attention.py '20200324141024(wormmouse_sessnum0)'
       '12000' 'normalized_dataset/normalize_each' 'trans-norm' 'trans-dop3'
```

```
python get_attention.py '20200324141024(wormmouse_sessnum0)'
       '12000' 'normalized_dataset/normalize_each' 'normal-pre_sm2' 'pd-pre_sm2'
```

'20200324141024(wormmouse_sessnum0)' is the name of the session. '12000' is the epoch number of the model used to output the attention values. It is possible that the network training is unstable. Therefore, please select the number that gives high class accuracy and poor domain accuracy by referring to the result in the 'accuracy' directory.

3. Training decision trees

```
python decision_tree_check.py '20200324141024(wormmouse_sessnum0)_step-12000'
       'attention-layer' 'various_feature' 'attention_speed_high' 'classifier' '2'
```

```
python decision_tree_check.py '20200324141024(wormmouse_sessnum0)_step-12000'
       'attention-layer' 'various_feature_speed_ft_xmean' 'normal_prob_classify' 'classifier' '2'
```

'20200324141024(wormmouse_sessnum0)_step-12000' indicates the name of session and the epoch number used. The first command generates a decision tree for detecting attended segments and the second command generates a decision tree for classifying each trajectory. '2' specifies the tree depth. When running this command, the user is required to input the following information.
domainA/classA: Please input the 1st class name of a domain of interest (e.g., 'trans-norm').
domainA/classB: Please input the 2nd class name of the domain (e.g., 'trans-dop3').

domainA_name: Please input the name of the domain (e.g., ’worm’).
This command stores resulting decision trees in ’analysis_set/(session name)/decisiontree_check’.

4. Generating graphs

```
python plot_highlight.py ’20200324141024(wormmouse_sessnum0)_step-12000’ ’original’
```

```
python show_images.py ’20200324141024(wormmouse_sessnum0)_step-12000’ ’svg’ ’8’
       ’attention_highlight’ ’svg_output’
```

The first command generates highlighted trajectories/time-series in ’analysis_set/(session name)/highlighted_trajectory’. The second command generates HTML files that show the generated images in ’analysis_set/(session name)/highlighted_trajectory_view’.

Supplementary Figures for Cross-species Behavior Analysis with Attention-based Domain-adversarial Deep Neural Networks

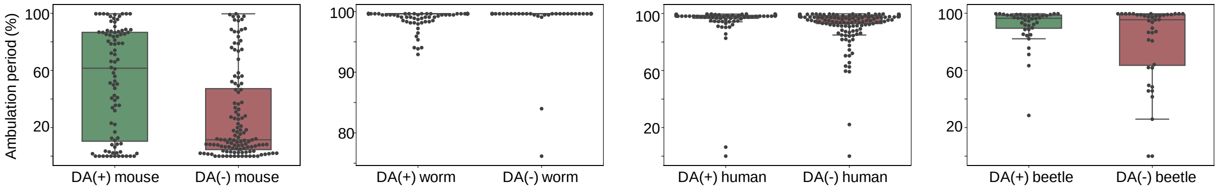

Supplementary Figure 1. Distributions of ambulation period[1]. The ambulation period is defined as periods when the velocity of an animal is higher than 2 cm/s for at least 0.5 s and is used to evaluate PD symptom of mice. We used this feature because it can be calculated from a speed time-series but trajectory. (We only have speed time-series of humans.) Note that parameters used to calculate this feature depend on animal species. We used grid search to find the best parameters (speed and time parameters) that give the largest difference in the averaged ambulation periods between DA(+) and DA(-). Because the speed parameter used in the mouse study (2 cm/s) was about 3.4% of the maximum speed of the mice in our data set, we have set the upper limit of the scan range of the grid search to be 10% of the maximum speed of each species. We could observe significant differences between DA(+) and DA(-) for the mice and beetles by the Welch's t-test (mouse: $p = 1.28 \times 10^{-6}$: $t = 5.02$; $\mathrm{df} = 169.28$; $\mathrm{effect\ size(r)} = -0.71$; beetle: $p = 0.03$; $t = 2.23$; $\mathrm{df} = 55.75$; $\mathrm{effect\ size(r)} = -0.50$). The p-value is two sided. However, we could not observe significant differences between DA(+) and DA(-) for the humans and worms (human: $p = 0.39$: $t = 0.86$; $\mathrm{df} = 129.18$; $\mathrm{effect\ size(r)} = -0.12$; worm: $p = 0.40$; $t = 0.84$; $\mathrm{df} = 48.00$; $\mathrm{effect\ size(r)} = -0.17$). The best speed and time parameters are 0.5 cm/s and 0 s for mice, 0.008 mm/s and 8 s for worms, 0.06 mm/s and 8s for beetles, and 3 and 9 for humans. (Note that speed time-series of humans do not have a unit.) $n = 78$ from different DA(+) humans. $n = 163$ from different DA(-) humans. $n = 88$ 10-min trajectories from DA(+) mice. $n = 113$ 10-min trajectories from DA(-) mice. $n = 162$ from different DA(+) worms. $n = 47$ from different DA(-) worms. $n = 40$ from different DA(+) beetles. $n = 40$ from different DA(-) beetles. The box plot shows the 25-75% quartile, with embedded bar representing the median and the lower/upper whiskers show Q1-1.5*IQR and Q3+1.5*IQR, respectively, where IQR is the interquartile range, Q1 is 25% quartile, and Q3 is 75% quartile.

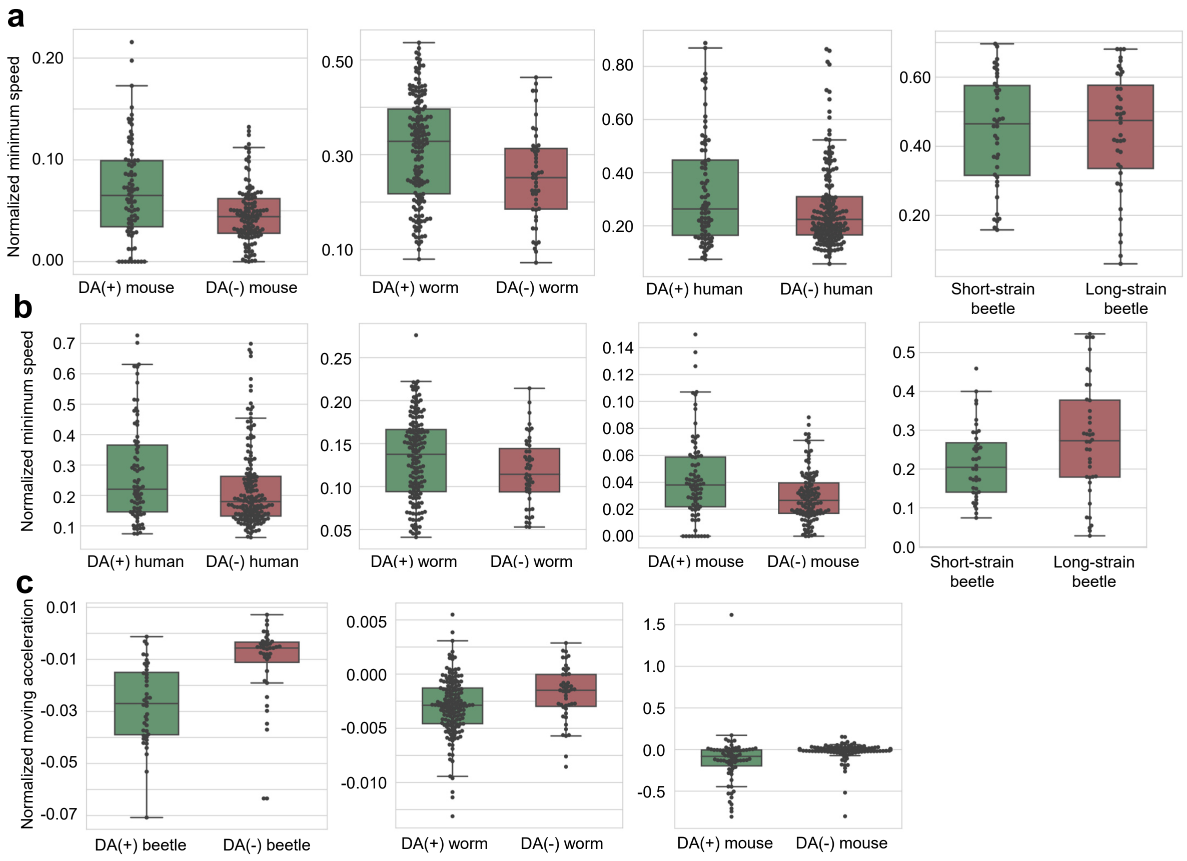

Supplementary Figure 2. Distributions of locomotion features found in our experiment. **a**, Distributions of mice, worms, humans, and beetles for the hypothesis obtained when training the network on data from mice and worms. **b**, Distributions of humans, worms, mice, and beetles for the hypothesis obtained when training the network on data from humans and worms. **c**, Distributions of beetles, worms, and mice for the hypothesis obtained when training the network on data from beetles and worms. $n = 78$ from different DA(+) humans. $n = 163$ from different DA(-) humans. $n = 88$ 10-min trajectories from DA(+) mice. $n = 113$ 10-min trajectories from DA(-) mice. $n = 162$ from different DA(+) worms. $n = 47$ from different DA(-) worms. $n = 40$ from different DA(+) beetles. $n = 40$ from different DA(-) beetles. The box plot shows the 25-75% quartile, with embedded bar representing the median and the lower/upper whiskers show Q1-1.5*IQR and Q3+1.5*IQR, respectively, where IQR is the interquartile range, Q1 is 25% quartile, and Q3 is 75% quartile.

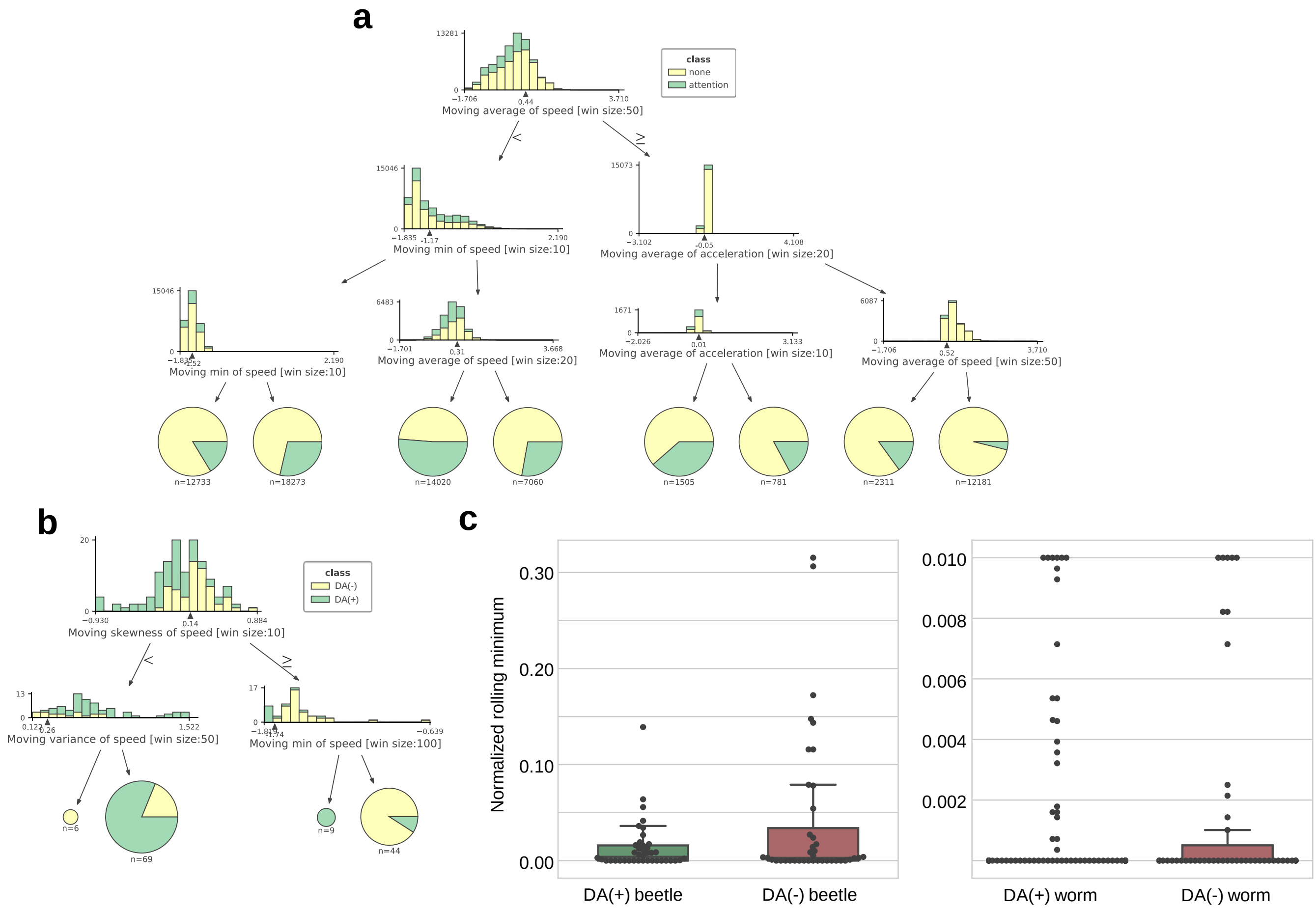

Supplementary Figure 3. Analysis on the network trained on data from beetles and worms using decision trees. **a**, A decision tree for explaining the meaning of attention. **b**, A decision tree for explaining classification. **c**, Distributions of rolling minimum of speed when the speed is slow for beetles and worms. $n = 162$ from different DA(+) worms. $n = 47$ from different DA(-) worms. $n = 40$ from different DA(+) beetles. $n = 40$ from different DA(-) beetles. The box plot shows the 25-75% quartile, with embedded bar representing the median and the lower/upper whiskers show Q1-1.5*IQR and Q3+1.5*IQR, respectively, where IQR is the interquartile range, Q1 is 25% quartile, and Q3 is 75% quartile.

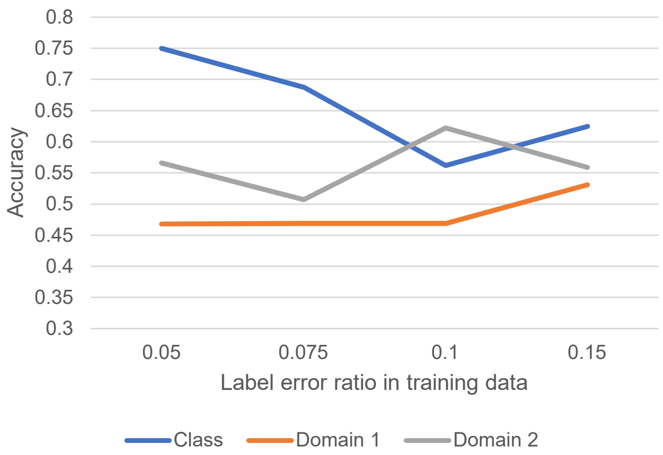

Supplementary Figure 4. Transitions of classification accuracies for DA(+) vs. DA(-), 1st domain estimates, and 2nd domain estimates in the study of the worms and mice when erroneous class labels of training trajectories are included

a

Estimates of Domain 1 in training

	DA(+)	DA(-)
Worm	1.0	1.0
Mouse	0.06	0.16

Estimates of Domain 1 in testing

	DA(+)	DA(-)
Worm	1.0	1.0
Mouse	0.0	0.0

Estimates of Domain 2 in training

	DA(+)	DA(-)
Worm	0.23	0.17
Mouse	0.88	0.89

Estimates of Domain 2 in testing

	DA(+)	DA(-)
Worm	0.23	0.15
Mouse	0.84	0.88

b

Estimates of Domain 1 in training

	DA(+)	DA(-)
Worm	0.0	0.81
Human	0.97	0.37

Estimates of Domain 1 in testing

	DA(+)	DA(-)
Worm	0.0	0.38
Human	0.63	0.38

Estimates of Domain 2 in training

	DA(+)	DA(-)
Worm	1.0	1.0
Human	0.05	0.05

Estimates of Domain 2 in testing

	DA(+)	DA(-)
Worm	1.0	1.0
Human	0.05	0.05

c

Estimates of Domain 1 in training

	DA(+)	DA(-)
Worm	0.0	0.88
Beetle	1.0	0.44

Estimates of Domain 1 in testing

	DA(+)	DA(-)
Worm	0.38	0.75
Beetle	0.63	0.63

Estimates of Domain 2 in training

	DA(+)	DA(-)
Worm	1.0	1.0
Beetle	0.0	0.0

Estimates of Domain 2 in testing

	DA(+)	DA(-)
Worm	1.0	1.0
Beetle	0.0	0.0

Supplementary Figure 5. Classification accuracies of our network for the 1st and 2nd domain estimates for each domain and class: **a**) domain classification accuracies for the analysis of worms and mice; **b**) domain classification accuracies for the analysis of worms and humans; **c**) domain classification accuracies for the analysis of worms and beetles

Supplementary Tables for Cross-species Behavior Analysis with Attention-based Domain-adversarial Deep Neural Networks

Supplementary Table 1. Features used in decision tree learning

Feature	Description
speed	Speed based on time unit
acceleration	Acceleration based on time unit
moving average of speed	Calculated within a time window of 10, 20, 50, 100 samples
moving variance of speed	Calculated within a time window of 10, 20, 50, 100 samples
moving skewness of speed	Calculated within a time window of 10, 20, 50, 100 samples
moving kurtosis of speed	Calculated within a time window of 10, 20, 50, 100 samples
maximum speed within a time window	Calculated within a time window of 10, 20, 50, 100 samples
minimum speed within a time window	Calculated within a time window of 10, 20, 50, 100 samples
moving average of acceleration	Calculated within a time window of 10, 20, 50, 100 samples
moving variance of acceleration	Calculated within a time window of 10, 20, 50, 100 samples
moving skewness of acceleration	Calculated within a time window of 10, 20, 50, 100 samples
moving kurtosis of acceleration	Calculated within a time window of 10, 20, 50, 100 samples
maximum acceleration within a time window	Calculated within a time window of 10, 20, 50, 100 samples
minimum acceleration within a time window	Calculated within a time window of 10, 20, 50, 100 samples

Supplementary Table 2. Overview of data sets. To train/test the network, 40 trajectories from each class are used. All trajectories are used in statistical tests.

Domain	1st class	2nd class	# trajectories (1st)	# trajectories (2nd)	Sampling rate	Avg. duration of trajectories	Avg. data length (data points)	Collection
worm (*Caenorhabditis elegans*)	normal	*dop-3*	162	47	1 Hz	540s	540	camera
mouse (*Mus musculus*)	normal	PD	88	113	6.9 Hz	78s	540	camera
human	normal	PD	78	163	6.7Hz	81s	540	gait sensor
red flour beetle (*Tribolium castaneum*)	S-strain	L-strain	40	40	1.3Hz	415s	540	treadmill

Supplementary Table 3. ANOVA F-values of features for detecting attended segments in the analysis of worms and mice (top-5 features) calculated by using scipy (v.1.2.1)

Feature	ANOVA F-value	effect size (η^2)
Moving average of speed [win size:20]	7832.6	0.185
Moving average of speed [win size:10]	7592.1	0.181
Moving min of speed [win size:10]	7277.1	0.174
Moving min of speed [win size:20]	5912.8	0.147
Moving average of speed [win size:50]	3941.2	0.103

Supplementary References for Cross-species Behavior Analysis with Attention-based Domain-adversarial Deep Neural Networks

References

1. Kravitz, A. V. *et al.* Regulation of parkinsonian motor behaviors by optogenetic control of basal ganglia circuitry. *Nature* **466**, 622–626 (2013).
