# Supplementary material for: Cross-species behavior analysis with attention-based domain-adversarial deep neural networks
Source: Nat Commun. 2021 Sep 17;12:5519. doi: 10.1038/s41467-021-25636-x (PMC8448872; doi:10.1038/s41467-021-25636-x)
Supplement: Supplementary file 2 — Description of Additional Supplementary Files [file 41467_2021_25636_MOESM2_ESM.pdf]

Description of Additional Supplementary Files

File Name: Supplementary Software 1
Description: Supplementary Software and Data
